# Orthostatic blood pressure adaptations, aortic stiffness, and central hemodynamics in the general population: insights from the Malmö Offspring Study (MOS)

**DOI:** 10.1007/s10286-022-00911-z

**Published:** 2022-12-06

**Authors:** Madeleine Johansson, Artur Fedorowski, Jens Jordan, Gunnar Engström, Peter M. Nilsson, Viktor Hamrefors

**Affiliations:** 1 Clinical Research Center, Department of Clinical Sciences-Hypertension and Cardiovascular Disease, Lund University, P.O. Box 50332, 202 13 Malmö, Sweden; 2 Department of Cardiology, Skåne University Hospital, Malmö, Sweden; 3 Department of Cardiology, Karolinska University Hospital, Stockholm, Sweden; 4 Department of Medicine, Karolinska Institute, Stockholm, Sweden; 5 Institute of Aerospace Medicine, German Aerospace Center, Cologne, Germany; 6 Medical Faculty, University of Cologne, Cologne, Germany

**Keywords:** Aortic stiffness, Arterial stiffness, Blood pressure, Epidemiology, Orthostatic hypotension, Population study, Vascular aging

## Abstract

**Purpose:**

Arterial stiffness is independently associated with orthostatic hypotension in older individuals. The relationship between orthostatic blood pressure adaptation and aortic stiffness has not been thoroughly examined in a younger population. We investigated the relationship between orthostatic blood pressure adaptations, central aortic hemodynamics, and aortic stiffness in a cohort of predominantly younger and middle-aged adults.

**Methods:**

We analyzed an observational, population-based study of 5259 individuals living in Malmö, Sweden. We related aortic stiffness and central hemodynamics assessed by carotid–femoral pulse wave velocity and pulse wave analysis at the arteria radialis using Sphygmocor to orthostatic blood pressure adaptation after 3 min standing.

**Results:**

The mean age of the population was 41.9 ± 14.5 years, and 52.1% were women. We observed the highest aortic stiffness and central aortic blood pressure measurements in the lowest and highest quartiles of orthostatic systolic blood pressure differences (*p* < 0.001). Aortic stiffness and central aortic blood pressure gradually decreased across increasing quartiles of orthostatic diastolic blood pressure difference (*p* < 0.001). After full adjustment, orthostatic diastolic blood pressure remained significantly associated with aortic stiffness (*p* = 0.001) and central aortic blood pressure (*p* < 0.001), whereas orthostatic systolic blood pressure was significantly associated only with central aortic systolic blood pressure (*p* = 0.009). No significant associations were found between subclinical orthostatic hypotension, aortic stiffness, and central hemodynamics.

**Conclusions:**

Our findings demonstrate that altered blood pressure responses to orthostatic challenges, both blood pressure reductions and blood pressure increases, are independently and inversely associated with markers of aortic stiffness (vascular aging) in a predominantly young to middle-aged population.

**Supplementary Information:**

The online version contains supplementary material available at 10.1007/s10286-022-00911-z.

## Introduction

Standing up imposes a major hemodynamic burden such that abnormalities in autonomic cardiovascular control or in cardiovascular structure may be unmasked. Therefore, orthostatic testing in addition to diagnosing autonomic cardiovascular impairments provides information regarding cardiovascular risk. Indeed, orthostatic hypotension (OH) defined as a decrease in brachial systolic blood pressure (SBP) of ≥ 20 mmHg and/or a decrease in diastolic blood pressure (DBP) of 10 mmHg or more within 3 min upon standing [1], independently predicts falls, mortality, and cardiovascular diseases in older adults [2–4]. Moreover, abnormalities in orthostatic blood pressure regulation including abnormal SBP decrease ≥ 20 mmHg [5] or increase ≥ 20 mmHg [6] upon standing, a so-called orthostatic hypertension, as well as abnormal BP variability [7] may be associated with cardiovascular disease and death. However, considerably fewer data exist regarding potential cardiovascular risks associated with subclinical orthostatic BP abnormalities, which are more common than full-blown orthostatic hypo- or hypertension, especially in younger persons.

Even in large-scale epidemiological studies, extremely long follow-up periods would be required to show an association between putative cardiovascular risk factors and morbidity and mortality in younger persons. Aortic stiffness mirrors structural changes in the aortic wall and adjacent large arteries and is, therefore, an established vascular aging marker in the setting of epidemiological studies and in the clinic [8–11]. Noninvasive measurement of carotid–femoral pulse wave velocity (c-f PWV) allows direct quantification of aortic stiffness and is considered the gold-standard method [11]. Aortic augmentation index (AIx) is based on pulse-wave reflection to the heart, and is an indirect measure of aortic stiffness [12]. Both c-f PWV and OH predict future cardiovascular events in middle-aged and older subjects [3, 13]. However, considerably fewer data are available on the potential relationships between subtle abnormalities in BP homeostasis and early markers of vascular aging in younger subjects.

In a Swedish population-based cohort, we tested the hypothesis that orthostatic BP responses are associated with vascular aging markers in younger to middle-aged persons.

## Methods

### Study population and design

The Malmö Offspring Study (MOS) is a population-based study consisting of children and grandchildren to the index participants in the well-established Malmö Diet and Cancer Study (MDCS) launched in the early 1990s with over 20 years of follow-up [14]. The data collection within MOS started in 2013 and was completed by the end of 2021. To ensure that participants in the different generations were directly related, a national taxation authority register in Sweden (NAVET) was used. A total of 5259 individuals were included in MOS. Of these, 3966 individuals had complete recordings of measurements of orthostatic BP reactions, aortic stiffness, and central hemodynamics, and were thus included in the final study population.

### Outcome measures

The primary outcome was increased aortic stiffness (c-f PWV) in relation to orthostatic BP differences. Secondary outcomes included increased aortic stiffness and BP reaction on standing in relation to sex and increasing age, respectively.

### Assessment of blood pressure, vascular aging, and aortic stiffness

Supine systolic (SBP) and diastolic blood pressure (DBP) were reported after 5 min rest as the mean of two readings, using an automatic device (Omron M5-1 IntelliSense, the Netherlands). Subjects were thereafter asked to stand up, and the mean SBP and DBP of two consecutive readings performed after 3 min of standing was reported as the orthostatic blood pressure. Aortic stiffness was directly assessed noninvasively after 5 min of supine rest using the gold-standard measurement of c-f PWV by Sphygmocor (AtCor, Australia) [15]. The distance from the carotid to femoral artery was measured directly between each artery and the suprasternal notch. PWV was calculated by measuring the time delay between two characteristic timepoints on two pressure waveforms at a known distance apart. The SphygmoCor method uses the foot of the waveform as an onset point for calculating the time differences between the R wave of the electrocardiogram and the pulse waveforms at each site. PWV was automatically generated as the carotid–femoral artery distance divided by the wave traveling time between the above two sites.

Aortic augmentation index (AIx), an indirect measurement of aortic stiffness, was together with variables reflecting central aortic hemodynamics (central aortic SBP and central aortic DBP) acquired by radial applanation tonometry using PWA Sphygmocor (AtCor, Australia). SphygmoCor also measures and calculates the AIX standardized to a heart rate of 75 beats per minute (AIx@75), by adjusting the AIX by −4.8 for each 10 bpm above and +4.8 for each 10 bpm below a resting heart rate of 75 bpm.

Brachial SBP and DBP were used to calibrate the radial and aortic pressure waveform. This technology has good reproducibility under major hemodynamic changes, analyzing a central (ascending aortic) pressure waveform from the radial pressure waveform using a validated generalized transfer function [16].

### Definition of variables and clinical characteristics

Data on prevalent diseases at baseline were collected from The Swedish National Patient Register. “Orthostatic BP difference” (mmHg) was defined as [standing BP − supine BP], i.e., a positive value denotes an increase in BP upon standing. Antihypertensive treatment at baseline was defined as self-reported intake of antihypertensive drugs. Estimated glomerular filtration rate (eGFR) was calculated using the CKD-EPI Creatinine Equation [17]. Current smoking (defined as regular or occasional smoking) and educational level were self-reported in the questionnaire.

### Ethical approval

The MOS study was approved by the Regional Ethics Committee at Lund University (Dnr. 2012/594). All participants provided written informed consent.

### Statistical analyses

Continuous data (of which all were normally distributed) are shown as mean ± standard deviation, whereas frequencies are used to describe categorical data. We first assessed aortic stiffness and central hemodynamic measurements by quartiles of orthostatic BP differences using one-way analysis of variance (ANOVA). Then, a linear regression model was constructed to assess the association of standing BP differences and subclinical orthostatic hypotension with aortic stiffness and central hemodynamics after different adjustments. The basic model was adjusted for age and sex. The multivariable model was adjusted for age, sex, body mass index (BMI), current smoking, fasting glucose, eGFR, antihypertensive medication, and supine SBP/DBP, as appropriate. Orthostatic diastolic BP reaction and aortic stiffness displayed a linear relationship, and was additionally adjusted for supine diastolic BP. In contrast, orthostatic systolic BP reaction displayed a U-shaped association with the outcome variables and was entered as mean-centered orthostatic systolic BP and adjusted for mean-centered supine systolic BP in addition to remaining covariates. For the secondary outcomes, analyses were performed separately according to sex and median age 44 years, respectively.

All statistical analyses were performed in IBM SPSS Statistics 27 (IBM Corporation, Armonk, NY, USA).

## Results

Clinical characteristics of the study population are presented in Table 1. The mean age was 42.1 ± 14.4 years (median 44 years, range 18–73 years), and 51.8% were women. A total of 14.2% reported current smoking, and less than 5% were diagnosed with prevalent hypertension and reported intake of antihypertensive medications. Following orthostatic blood pressure measurements, 1.3% fulfilled the diagnostic criteria of OH and 1.7% showed an orthostatic SPB increase of ≥ 20 mmHg, which is commonly used as diagnostic cutoff value for orthostatic hypertension [6].

**Table 1:** Baseline characteristics of the study population

	Total population (*n* = 3966)	Below 44 years old (*n* = 1952)	Above or equal to 44 years old (*n* = 2014)
Age (years ± SD)	42.1 ± 14.4	29.2 ± 7.4	54.6 ± 6.3
(Age range)	(18–73)	(18–43)	(44–73)
Sex, women (%)	51.8	52.2	51.7
BMI (kg/m^2^ ± SD)	26.0 ± 4.6	25.0 ± 4.4	27.1 ± 4.7
Current smoking (%)	14.2	17.6	11.5
Educational level (%)			
Less than 8 years	0.3	0.3	0.2
Elementary school	5.6	3.8	7.5
High school degree	50.8	54.8	47
University degree	33.6	30.8	35.8
Fasting glucose (mmol/L ± SD)	5.4 ± 1.1	5.2 ± 0.8	5.6 ± 1.3
eGFR (mL/min/1.73 m^2^)	83.5 ± 8.9	87.9 ± 5.5	79.2 ± 9.7
Antihypertensive treatment (%)	4.9	1	8.9
Prevalent disease (%)			
Atrial fibrillation	1	0.3	1.9
Diabetes mellitus	4	1.8	6.3
Hypertension	4.7	0.5	9.1
Orthostatic hypotension	1.3	0.6	2.1
Orthostatic hypertension	1.7	0.8	2.6
Blood pressure (mmHg ± SD)			
SBP, supine	118.9 ± 16.0	111.1 ± 11.7	126.6 ± 16.1
DBP, supine	73.9 ± 10.1	68.8 ± 7.9	79.0 ± 9.6
Orthostatic SBP reaction*	1.3 ± 8.6	1.38 ± 7.3	1.32 ± 9.7
Orthostatic DBP reaction*	9.5 ± 6.1	11.1 ± 5.8	7.9 ± 6.1
Central hemodynamic measurements			
c-f PWV (m/s ± SD)	7.5 ± 1.6	6.5 ± 1.0	8.3 ± 1.6
Aix (mean % ± SD)	8.4 ± 14.8	− 1.1 ± 11.8	17.8 ± 11.0
Aix@75 (mean % ± SD)	9.6 ± 74.4	− 0.9 ± 11.8	17.9 ± 11.0
Central aortic SBP (mmHg ± SD)	103.3 ± 16.1	94.2 ± 9.7	112.4 ± 16.1
Central aortic DBP (mmHg ± SD)	72.5 ± 9.9	67.5 ± 7.7	77.4 ± 9.4

*Aix* augmentation index, *Aix@75* augmentation index adjusted for a standard heart rate of 75 bpm, *BMI* body mass index, *BP* blood pressure, *c-f* carotid–femoral, *DBP* diastolic blood pressure, *eGFR* estimated glomerular filtration rate, *HR* heart rate, *PWV* pulse wave velocity, *SBP* systolic blood pressure, *SD* standard deviation

*Orthostatic BP reaction is defined as the difference between standing BP and supine BP

**Fig. 1:**
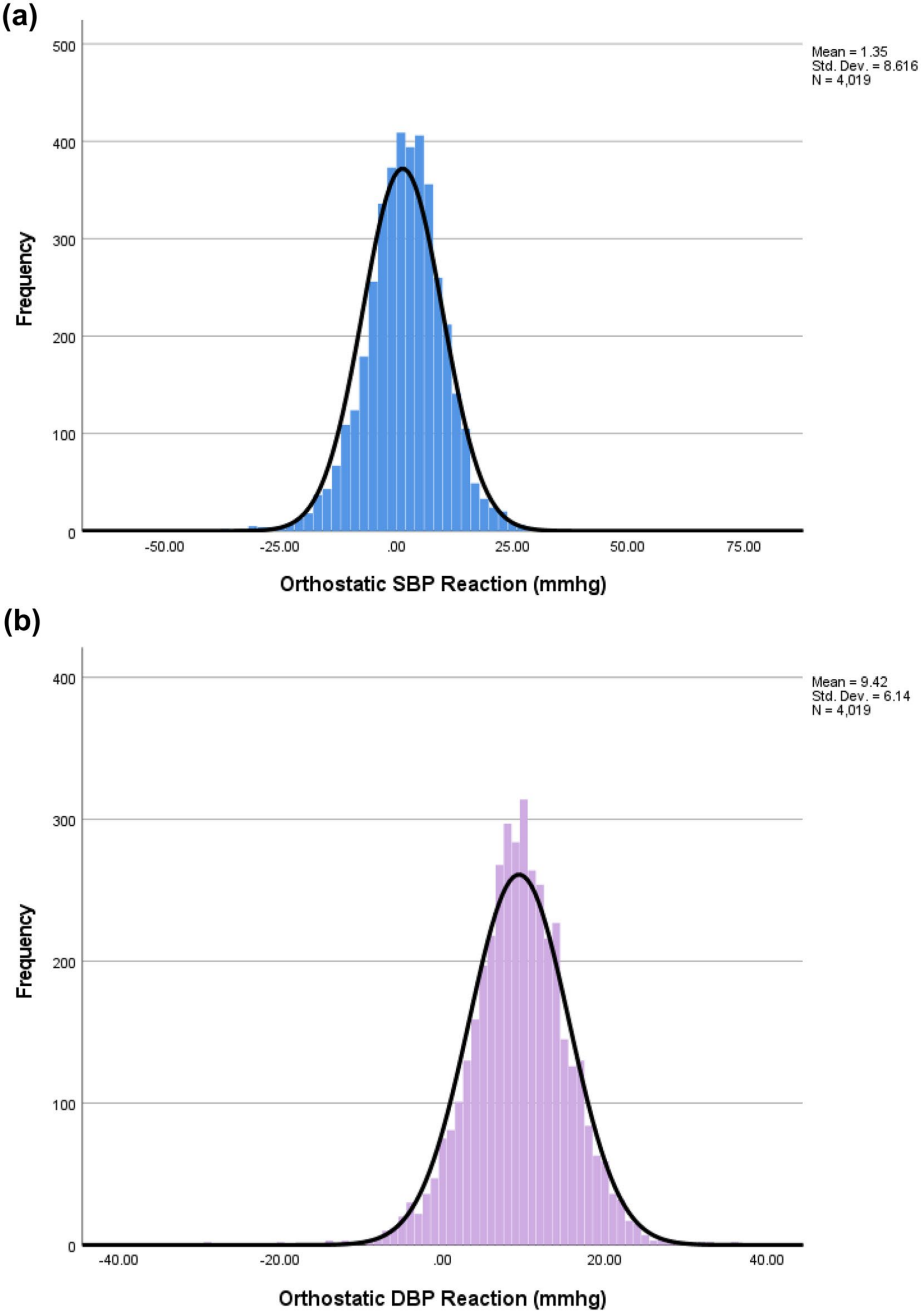
Distribution of orthostatic blood pressure reactions in the population. **A** Orthostatic systolic blood pressure. **B** Orthostatic diastolic blood pressure

The mean orthostatic SBP difference upon standing was 1.3 ± 8.6 mmHg, whereas the corresponding orthostatic DBP difference was 9.5 ± 6.1 mmHg. Orthostatic blood pressure reactions were all normally distributed (Fig. 1). Mean aortic stiffness measurements are provided in Table 1.

### Association between aortic stiffness, central hemodynamics, and orthostatic BP reactions

**Fig. 2:**
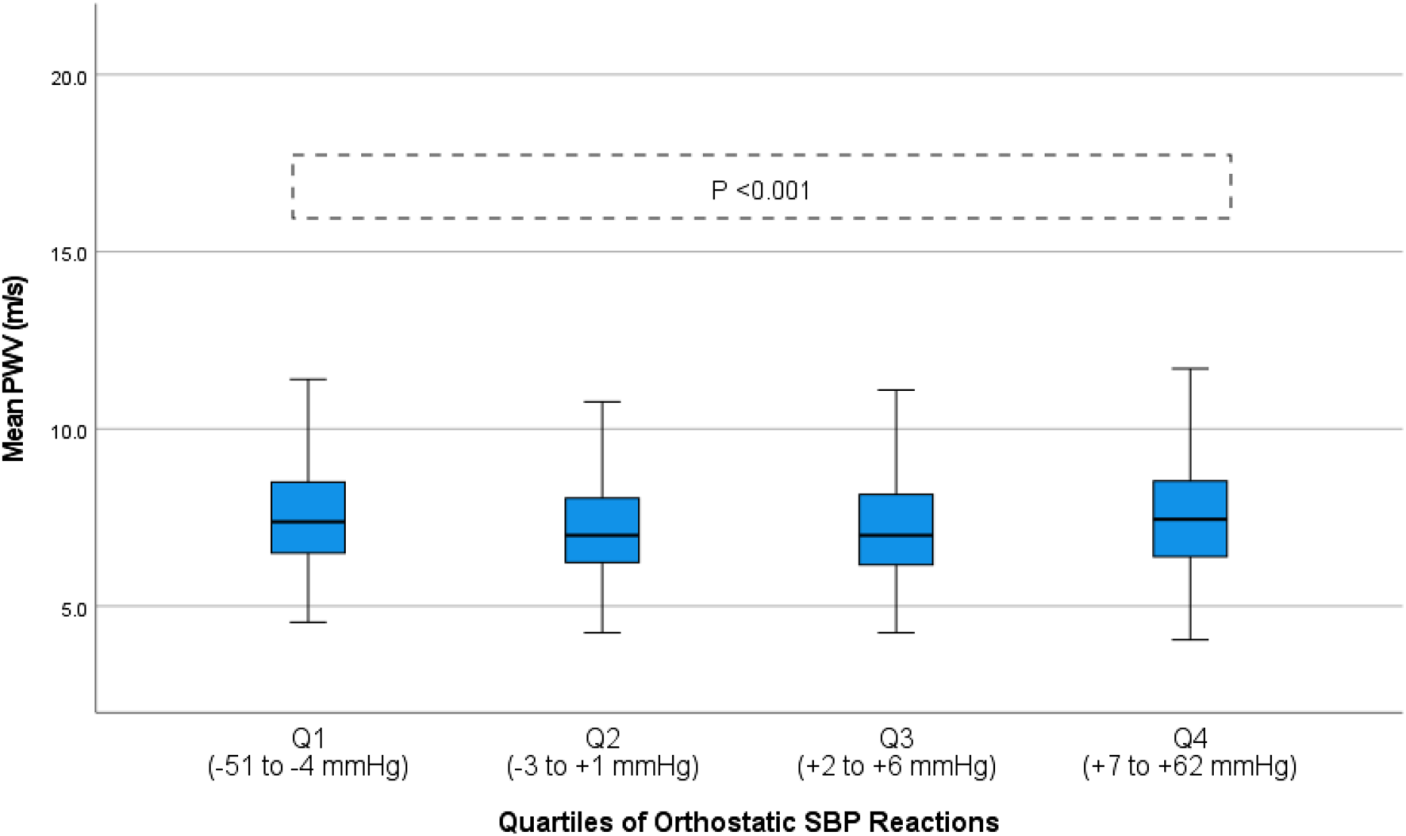
Aortic stiffness measured by pulse wave velocity stratified according to quartiles of orthostatic systolic blood pressure reaction. Boxplot illustrating aortic stiffness stratified according to quartiles of orthostatic systolic blood pressure reaction in the general population with reported ANOVA *p* value

**Fig. 3:**
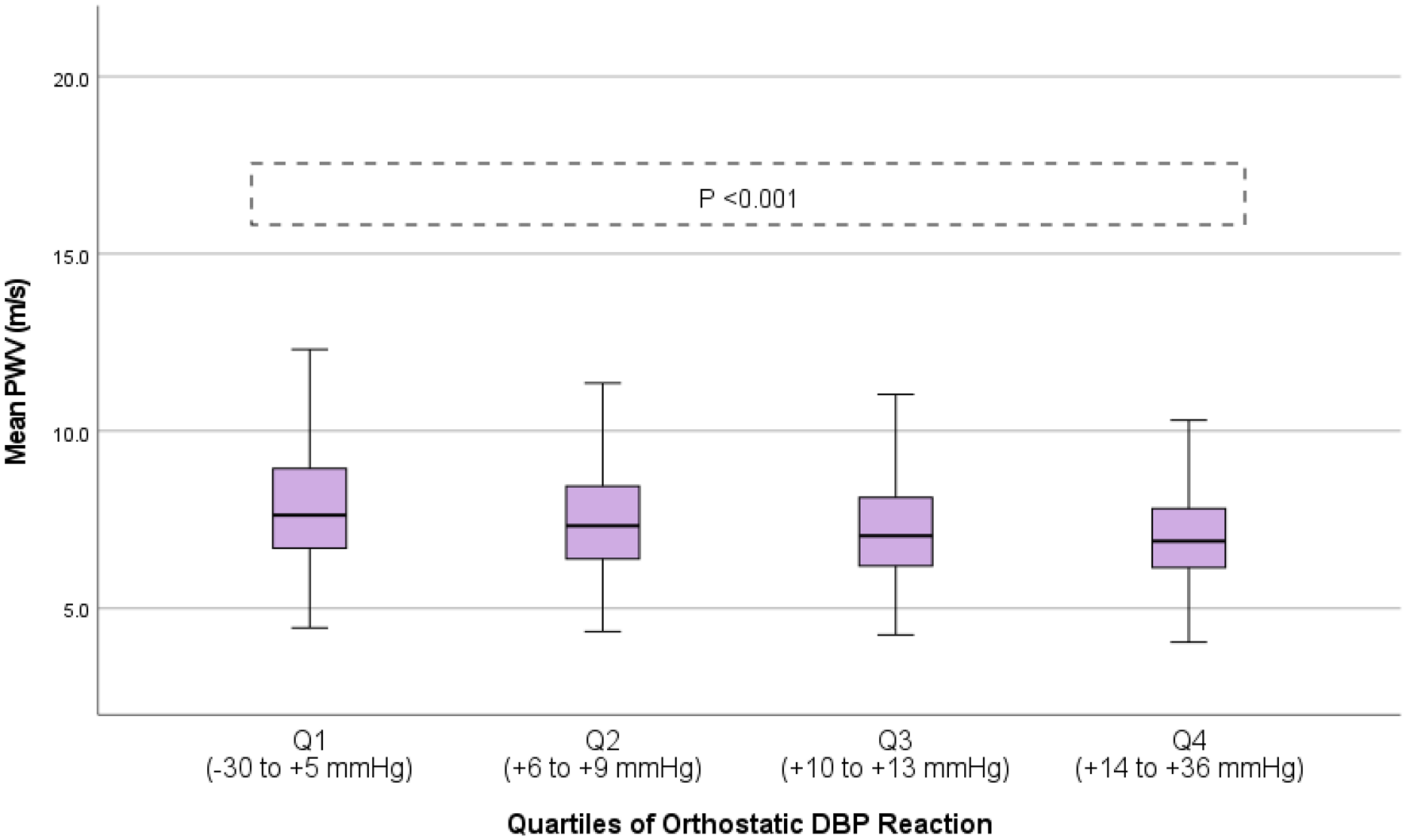
Aortic stiffness measured by pulse wave velocity stratified according to quartiles of orthostatic diastolic blood pressure reaction. Boxplot illustrating aortic stiffness stratified according to quartiles of orthostatic systolic blood pressure reaction in the general population with reported ANOVA *p* value

We observed a U-shaped association between orthostatic systolic BP differences, aortic stiffness, and central hemodynamics, i.e., subjects in the lowest and highest quartiles of orthostatic systolic BP differences demonstrated the highest degree of aortic stiffness and central aortic BP (all *p* < 0.001 in ANOVA; Fig. 2, Supplementary Fig. S1, Table 2). In contrast, orthostatic diastolic BP reaction displayed a linear association, with a gradual reduction in aortic stiffness and central aortic BP across all four quartiles of increasing orthostatic diastolic BP reaction (all *p* < 0.001 in ANOVA; Fig. 3, Supplementary Fig. S2, Table 2). No difference was observed in the sensitivity analysis after exclusion of subjects ≥ 60 years old (*n* = 372, 9.4%) (Supplementary Table S1).

**Table 2:** Aortic stiffness and central hemodynamics stratified according to quartiles of orthostatic systolic (SBP) and diastolic blood pressure (DBP) reaction in the entire general population (*n* = 3966)

Orthostatic SBP reaction	Mean ± SD	*p* value	Orthostatic DBP reaction	Mean ± SD	*p* value
Direct aortic stiffness—c-f PWV (m/s)					
Q1 (−51 to −4 mmHg)	7.7 ± 1.7	P < 0.001	Q1 (− 30 to +5 mmHg)	8.0 ± 1.8	*p* < 0.001
Q2 (−3 to +1 mmHg)	7.3 ± 1.5		Q2 (+6 to +9 mmHg)	7.5 ± 1.6	
Q3 (+2 to +6 mmHg)	7.3 ± 1.5		Q3 (+10 to +13 mmHg)	7.3 ± 1.5	
Q4 (+7 to +62 mmHg)	7.6 ± 1.6		Q4 (+14 to +36 mmHg)	7.1 ± 1.4	
Indirect aortic stiffness—AIx (mean %)					
Q1 (−51 to −4 mmHg)	9.5 ± 15.6	*p* < 0.001	Q1 (−30 to +5 mmHg)	14.2 ± 13.6	*p* < 0.001
Q2 (−3 to +1 mmHg)	7.2 ± 14.3		Q2 (+6 to +9 mmHg)	9.8 ± 14.6	
Q3 (+2 to +6 mmHg)	7.0 ± 14.7		Q3 (+10 to +13 mmHg)	6.8 ± 14.4	
Q4 (+7 to +62 mmHg)	10.3 ± 14.8		Q4 (+14 to +36 mmHg)	3.5 ± 14.6	
Indirect aortic stiffness—Aix@75 (mean %) adjusted for HR					
Q1 (−51 to −4 mmHg)	9.6 ± 15.6	*p* < 0.001	Q1 (−30 to +5 mmHg)	14.3 ± 13.7	*p* < 0.001
Q2 (−3 to +1 mmHg)	7.2 ± 14.2		Q2 (+6 to +9 mmHg)	9.9 ± 14.6	
Q3 (+2 to +6 mmHg)	6.9 ± 14.9		Q3 (+10 to +13 mmHg)	6.8 ± 14.3	
Q4 (+7 to +62 mmHg)	10.4 ± 14.2		Q4 (+14 to +36 mmHg)	3.6 ± 14.8	
Central hemodynamics—central aortic SBP (mmHg)					
Q1 (−51 to −4 mmHg)	106.3 ± 16.2	*p* < 0.001	Q1 (−30 to +5 mmHg)	109.2 ± 17.7	*p* < 0.001
Q2 (−3 to +1 mmHg)	101.1 ± 15.5		Q2 (+6 to +9 mmHg)	104.5 ± 16.0	
Q3 (+2 to +6 mmHg)	101.4 ± 15.3		Q3 (+10 to +13 mmHg)	101.2 ± 14.9	
Q4 (+7 to +62 mmHg)	104.7 ± 17.0		Q4 (+14 to +36 mmHg)	99.2 ± 16.2	
Central hemodynamics—central aortic DBP (mmHg)					
Q1 (−51 to −4 mmHg)	73.4 ± 10.0	*p* < 0.001	Q1 (−30 to +5 mmHg)	75.7 ± 10.1	*p* < 0.001
Q2 (−3 to +1 mmHg)	71.4 ± 9.9		Q2 (+6 to +9 mmHg)	73.5 ± 9.6	
Q3 (+2 to +6 mmHg)	71.6 ± 9.6		Q3 (+10 to +13 mmHg)	71.4 ± 9.7	
Q4 (+7 to +62 mmHg)	73.6 ± 10.2		Q4 (+14 to +36 mmHg)	69.8 ± 9.5	

*AIx* augmentation index, *Aix@75* augmentation index adjusted for a standard heart rate of 75 bpm, *c-f PWV* carotid–femoral pulse wave velocity, *DBP* diastolic blood pressure, *HR* heart rate, *Q* quartile, *SBP* systolic blood pressure, *SD* standard deviation

ANOVA analysis showing the association between aortic stiffness (i.e., c-f PWV, AIx, and AIx@75) and central hemodynamics (central aortic blood pressure) stratified according to quartiles of orthostatic blood pressure reactions

*p*-values are significant < 0.001

After full adjustments, orthostatic diastolic BP remained significantly associated with aortic stiffness (c-f PWV *p *= 0.001, Aix *p* = 0.005, and Aix@75 *p* = 0.007) and central aortic BP (*p* < 0.001), whereas orthostatic systolic BP was significantly associated only with indirect aortic stiffness adjusted for HR (Aix@75 *p* = 0.04) and central aortic systolic BP (*p* = 0.009) (data not shown). No significant associations were found between subclinical manifest orthostatic hypotension, aortic stiffness, and central hemodynamics (Supplementary Table S2).

### Subgroup analysis stratified by age and sex

We performed fully adjusted subgroup analysis of aortic stiffness and central hemodynamics stratified by median age (Table 3). Overall, orthostatic diastolic BP was inversely associated with indirect aortic stiffness (AIX@75) (*p* = 0.003 and *p* < 0.001, Table 3), and central aortic systolic as well as diastolic BP in both age groups (*p* < 0.001). Orthostatic systolic BP was associated only with indirect aortic stiffness in older individuals (Aix *p* = 0.008, Aix@75 *p* = 0.008), whereas it was inversely associated with central aortic systolic BP in both age groups (*p* = 0.008 versus *p* = 0.049). Manifest subclinical OH was associated with central aortic systolic BP in the above-median-age group only.

**Table 3:** Association between orthostatic blood pressure reaction, manifest orthostatic hypotension, and aortic stiffness as well as central hemodynamics stratified by median age

	Fully adjusted model	
	< 44 years old	≥ 44 years old
	*p* value	*p* value
Direct aortic stiffness (c-f PWV)		
Orthostatic SBP difference	0.21	0.13
Orthostatic DBP difference	** < 0.001**	0.61
Orthostatic hypotension	0.59	0.10
Indirect aortic stiffness (AIx)		
Orthostatic SBP difference	0.35	**0.008**
Orthostatic DBP difference	**0.005**	0.27
Orthostatic hypotension	0.95	0.61
Indirect aortic stiffness (AIx@75) adjusted for HR		
Orthostatic SBP difference	0.40	**0.006**
Orthostatic DBP difference	**0.003**	** < 0.001**
Orthostatic hypotension	0.91	0.39
Central hemodynamics (central aortic SBP)		
Orthostatic SBP difference	**0.008**	**0.049**
Orthostatic DBP difference	** < 0.001**	**0.001**
Orthostatic hypotension	0.76	**0.02**
Central hemodynamics (central aortic DBP)		
Orthostatic SBP difference	0.05	0.27
Orthostatic DBP difference	** < 0.001**	** < 0.001**
Orthostatic hypotension	0.68	0.31

Linear regression analysis showing the association between measures of aortic stiffness (c-f PWV, Aix, and Aix@75) and central hemodynamics (central aortic blood pressure) and orthostatic blood pressure reactions stratified by median age of 44 years. Fully adjusted model adjusted for sex, BMI, eGFR, fasting glucose, current smoking antihypertensive medications, and squared mean-centered supine SBP/supine DBP/supine SBP as appropriate, as further described in “Methods”

Significant* p* values are in bold

*AIx* augmentation index, *Aix@75* augmentation index adjusted for a standard heart rate of 75 bpm, *BMI* body mass index, *c-f PWV* carotid–femoral pulse wave velocity, *DBP* diastolic blood pressure, *eGFR* estimated glomerular filtration rate, *HR* heart rate, *OH* orthostatic hypotension, *SBP* systolic blood pressure

Following sex stratification, orthostatic diastolic BP was inversely associated with direct aortic stiffness (c-f PWV *p* = 0.03 versus *p* = 0.006, Table 4), and central aortic systolic and diastolic BP (all *p* < 0.001) in both sexes. Only women displayed an inverse association between aortic stiffness and orthostatic diastolic BP (AIx *p* = 0.03 and *p* = 0.04), and a positive association between orthostatic hypotension and central aortic systolic BP (*p* = 0.02). Likewise, central aortic systolic and diastolic BP was significantly associated only with orthostatic systolic BP in women (all *p* = 0.02).

**Table 4:** Association between orthostatic blood pressure reaction and manifest orthostatic hypotension, aortic stiffness, and central hemodynamics stratified by sex

	Fully adjusted model	
	Men	Women
	*p* value	*p* value
Direct aortic stiffness (c-f PWV)		
Orthostatic SBP difference	0.57	0.21
Orthostatic DBP difference	**0.03**	**0.006**
Orthostatic hypotension	0.46	0.06
Indirect aortic stiffness (AIx)		
Orthostatic SBP difference	0.37	0.14
Orthostatic DBP difference	0.10	**0.03**
Orthostatic hypotension	0.43	1.00
Indirect aortic stiffness (AIx@75) adjusted for HR		
Orthostatic SBP difference	0.48	0.06
Orthostatic DBP difference	0.09	**0.04**
Orthostatic hypotension	0.40	0.64
Central hemodynamics (central aortic SBP)		
Orthostatic SBP difference	0.28	**0.02**
Orthostatic DBP difference	** < 0.001**	** < 0.001**
Orthostatic hypotension	0.81	**0.005**
Central hemodynamics (central aortic DBP)		
Orthostatic SBP difference	0.99	**0.02**
Orthostatic DBP difference	** < 0.001**	** < 0.001**
Orthostatic hypotension	0.92	0.30

Linear regression analysis showing the association between aortic stiffness (c-f PWV, Aix, and Aix@75) and central hemodynamics (central aortic blood pressure) and orthostatic blood pressure reactions stratified by sex. Fully adjusted model adjusted for age, BMI, eGFR, fasting glucose, current smoking antihypertensive medications, and squared mean-centered supine SBP/supine DBP/supine SBP as appropriate, as further described in “Methods”

Significant* p* values are in bold

*AIx* augmentation index, *Aix@75* augmentation index adjusted for a standard heart rate of 75 bpm, *BMI* body mass index, *c-f PWV* carotid–femoral pulse wave velocity, *DBP* diastolic blood pressure, *eGFR* estimated glomerular filtration rate, *HR* heart rate, *OH* orthostatic hypotension diagnosis, *SBP* systolic blood pressure

## Discussion

In this population-based study of predominantly young to middle-aged subjects, we assessed the relationship between orthostatic BP adaptations and aortic stiffness as well as central aortic BP. We found that a more pronounced (higher) diastolic BP increase upon standing is associated with lower aortic stiffness (c-f PWV), whereas the systolic blood pressure reaction upon standing displayed a U-shaped association with aortic stiffness (c-f PWV).

To date, manifest orthostatic hypotension (OH) has been widely examined in older populations, and the prevalence of OH in the general population increases with age and comorbidities such as neurodegenerative, cardiovascular, metabolic, and renal disorders [18, 19]. Considerably fewer data exist for OH as well as the potential risks of subclinical blood pressure abnormalities in younger subjects.

In the current observational study, we provide evidence supporting the view that even subtle abnormalities in blood pressure reactions upon standing are associated with markers of increased vascular aging (arterial stiffness) in an otherwise predominantly young and healthy population.

### Results compared with previous studies

A distinct increase in diastolic BP of approximately 10 mmHg upon standing is considered the normal reaction [20], and accordingly we observed that persons with a diastolic BP increase on standing of ≥ 14 mmHg had the lowest aortic stiffness. This is in line with previous data from the Framingham Heart Study Third Generation cohort, even though mean arterial pressure (MAP) was used in that study [8]. For the systolic BP increase upon standing, an U-shaped association with aortic stiffness was noted, which is in line with data suggesting that both marked systolic blood pressure decrease [5] and increase [6] upon standing is associated with adverse cardiovascular outcomes [21].

Aortic stiffness is an important risk factor/marker for cardiovascular disease (CVD) and a key element in the pathogenesis of CVD. Growing evidence shows that increased aortic stiffness can predict both cardiovascular mortality and all-cause mortality [22, 23].

An epidemiological study such as ours cannot prove causality but may generate hypotheses for mechanism-oriented investigations. While we cannot discern whether altered orthostatic blood pressure control promoted vascular damage or vice versa, our study provides an impetus to investigate mutual interactions between cardiovascular autonomic control and vascular structure in more detail. For example, baroreflex counter-regulation, which stabilizes blood pressure upon standing, could be impeded by changes in vascular structure. In a previous study, we found that proteins associated with atherosclerosis were also related to impaired blood pressure control [19, 24]. Diminished baroreflexes often occur in patients with arterial stiffness due to impaired stretch of the baroreceptors and reduced neural input to the brain stem’s autonomic control centers, causing decreased output to the cardiovascular system. Another study demonstrated that asymptomatic subjects with subclinical coronary atherosclerosis display severely blunted baroreflexes and may also have advanced coronary atherosclerosis [25]. Conversely, blood pressure swings elicited through poor cardiovascular autonomic control could negatively affect vascular structure [7].

The current study, Malmö Offspring Study, is unique since it contains a wealth of data on younger and rather healthy individuals (mean age of 41 years) with few comorbidities. For example, the hypertension prevalence (based on use of antihypertensive drugs) was only 5%. In addition, this is the first population-based cohort to investigate the association of orthostatic BP reactions with well-established and validated assessments of aortic stiffness and central hemodynamics, including the gold-standard method c-f PWV, as well as central aortic hemodynamic measurements such as AIX and central aortic BP [26–28]. Previous studies have only assessed aortic stiffness by c-f PWV in this context [8, 21, 29–31] or brachial-to-ankle PWV [32]. Moreover, the abovementioned studies have focused on older individuals (mean age ranging from 44 to 80 years) with higher prevalence of established comorbidities such as type 2 diabetes or hypertension, ranging between 20% and 60%.

### Strengths and limitations of the study

To our knowledge, this is the first and largest observational study investigating the association between orthostatic blood pressure reactions and markers of aortic stiffness in nearly 4000 individuals with a mean age of 41 years. The Malmö Offspring (MOS) cohort was recently completed at the end of December 2021, providing unique data on study participants from national Swedish registers with complete coverage of public healthcare in Sweden, making our findings reliable and robust. Moreover, we assessed aortic stiffness by robust and validated methods with over 1000 peer-reviewed studies published reporting data derived from the well-validated tonometry-based SphygmoCor devices [33]. Our findings provide additional knowledge related to the potential importance of evaluating orthostatic BP adaptations at an early adult age in terms of risk assessment and in relation to markers of vascular aging. Future investigations in MOS will enable us to assess novel aspects of vascular aging, with the unique possibility of national register-based follow-up for morbidity and mortality.

However, our study has also some important limitations that need to be addressed. Study participants were predominantly young- to middle-aged individuals of white European ancestry, and therefore, the results of this study may not be generalizable to other racial/ethnic groups. Likewise, information on BP levels was obtained at a single visit, meaning that we were not able to assess the day-to-day reproducibility of our measurements. Furthermore, we could not draw any conclusions on causality from the cross-sectional design of the study, i.e., whether increased aortic stiffness leads to abnormal orthostatic BP reactions or vice versa, or if there is a common cause contributing to both aortic stiffness and abnormal orthostatic blood pressure reactions, for example, the influence of genetics or early life programming [34]. Since the MOS cohort was recently completed (end of 2021), we currently lack longitudinal data to perform prospective analyses.

### Study relevance and clinical implications

The co-existence of orthostatic hypotension, increased BP variability, and arterial stiffness represents a hemodynamic aging syndrome [10] with important prognostic implications for public health. These three entities are independent risk markers for CVD, and their confluence, therefore, is of impactful significance. In addition, increasing data indicate that also orthostatic hypertension confers an increased risk of CVD [6].

We demonstrate here that even subtle, subclinical abnormalities in orthostatic BP regulation are associated with changes in central hemodynamics and arterial stiffness—a marker of early vascular aging (EVA) [35]—in a population-based cohort of predominantly young adult and middle-aged healthy subjects, whereas the prevalence of subclinical OH was low. Only individuals above the median age of 44 years showed a positive correlation between a subclinical OH diagnosis and worsened central aortic hemodynamics, i.e., aortic stiffness, in the fully adjusted model, suggesting that clinical OH is likely a marker of more advanced vascular aging [30].

## Conclusions

Our findings support the hypothesis that impaired hemodynamic response to standing, traditionally observed in older individuals, is also associated with markers of vascular aging in a predominantly younger and healthy population. Further studies should assess the relationship between impaired blood pressure adaptations on standing in younger subjects and risk of future incident cardiovascular events. The clinical implication is that, in addition to diagnosing abnormalities in cardiovascular autonomic control, orthostatic testing may identify individuals at increased cardiovascular risk for preventive risk factor control.

## Supplementary Information

Below is the link to the electronic supplementary material.

Supplementary file1 (DOCX 156 KB)

## Data Availability

Anonymized data are available upon reasonable request to the corresponding author. Details on procedures can be found here: https://www.malmo-kohorter.lu.se/malmo-cohorts.
