# Supplementary material for: Orthostatic blood pressure adaptations, aortic stiffness, and central hemodynamics in the general population: insights from the Malmö Offspring Study (MOS)
Source: Clin Auton Res. 2022 Dec 6;33(1):29–40. doi: 10.1007/s10286-022-00911-z (PMC9984326; doi:10.1007/s10286-022-00911-z)
Supplement: Supplementary file 1 — Supplementary file1 (DOCX 156 KB) [file 10286_2022_911_MOESM1_ESM.docx]

**Supplement**

**Supplementary Tables.**

**Table S1. Aortic stiffness and central hemodynamics stratified according to quartiles of orthostatic systolic (SBP) and diastolic blood pressure (DBP) reaction in subjects 60 years and below (n=3594).**

| **Orthostatic**  **SBP reaction** | **Mean**  **± SD** | **P-value** | **Orthostatic**  **DBP reaction** | **Mean**  **± SD** | **P-value** |
| --- | --- | --- | --- | --- | --- |
| **DIRECT AORTIC STIFFNESS - c-f PWV (m/s)** | | | | | |
| **Q1**  **(-51 to -4 mmHg)** | 7.4 ± 1.5 | P <0.001 | **Q1**  **(-30 to +6 mmHg)** | 7.6 ± 1.6 | P <0.001 |
| **Q2**  **(-3 to +1 mmHg)** | 7.1 ± 1.4 |  | **Q2**  **(+7 to +9 mmHg)** | 7.3 ± 1.4 |  |
| **Q3**  **(+2 to +6 mmHg)** | 7.1 ± 1.4 |  | **Q3**  **(+10 to +13 mmHg)** | 7.1 ± 1.4 |  |
| **Q4**  **(+7 to +50 mmHg)** | 7.4 ± 1.4 |  | **Q4**  **(+14 to +36 mmHg)** | 7.0 ± 1.4 |  |
| **INDIRECT AORTIC STIFFNESS - AIx (mean %)** | | | | | |
| **Q1**  **(-51 to -4 mmHg)** | 7.4 ± 15.1 | P <0.001 | **Q1**  **(-30 to +6 mmHg)** | 11.6 ± 13.7 | P <0.001 |
| **Q2**  **(-3 to +1 mmHg)** | 5.9 ± 13.9 |  | **Q2**  **(+7 to +9 mmHg)** | 7.8 ± 14.3 |  |
| **Q3**  **(+2 to +6 mmHg)** | 5.5 ± 14.3 |  | **Q3**  **(+10 to +13 mmHg)** | 5.7 ± 14.2 |  |
| **Q4**  **(+7 to +50 mmHg)** | 8.3 ± 14.0 |  | **Q4**  **(+14 to +36 mmHg)** | 2.1 ± 13.8 |  |
| **INDIRECT AORTIC STIFFNESS – Aix@75 (mean %) adjusted for HR** | | | | | |
| **Q1**  **(-51 to -4 mmHg)** | 7.4 ± 15.1 | P <0.001 | **Q1**  **(-30 to +6 mmHg)** | 11.6 ± 13.7 | P <0.001 |
| **Q2**  **(-3 to +1 mmHg)** | 5.9 ± 13.8 |  | **Q2**  **(+7 to +9 mmHg)** | 7.9 ± 14.3 |  |
| **Q3**  **(+2 to +6 mmHg)** | 5.4 ± 14.5 |  | **Q3**  **(+10 to +13 mmHg)** | 5.6 ± 14.1 |  |
| **Q4**  **(+7 to +50 mmHg)** | 8.4 ± 14.0 |  | **Q4**  **(+14 to +36 mmHg)** | 2.1 ± 14.4 |  |
| **CENTRAL HEMODYNAMICS - Central Aortic SBP (mmHg)** | | | | | |
| **Q1**  **(-51 to -4 mmHg)** | 103.8 ± 14.4 | P <0.001 | **Q1**  **(-30 to +6 mmHg)** | 105.9 ± 16.3 | P <0.001 |
| **Q2**  **(-3 to +1 mmHg)** | 99.6 ± 14.4 |  | **Q2**  **(+7 to +9 mmHg)** | 102.0 ± 14.6 |  |
| **Q3**  **(+2 to +6 mmHg)** | 99.8 ± 14.4 |  | **Q3**  **(+10 to +13 mmHg)** | 100.0 ± 14.0 |  |
| **Q4**  **(+7 to +50 mmHg)** | 102.5 ± 15.8 |  | **Q4**  **(+14 to +36 mmHg)** | 97.7 ± 13.2 |  |
| **CENTRAL HEMODYNAMICS - Central Aortic DBP (mmHg)** | | | | | |
| **Q1**  **(-51 to -4 mmHg)** | 72.5 ± 9.6 | P <0.001 | **Q1**  **(-30 to +6 mmHg)** | 74.6 ± 9.9 | P <0.001 |
| **Q2**  **(-3 to +1 mmHg)** | 70.7 ± 9.6 |  | **Q2**  **(+7l to +9 mmHg)** | 72.7 ± 9.4 |  |
| **Q3**  **(+2 to +6 mmHg)** | 71.0 ± 9.5 |  | **Q3**  **(+10 to +13 mmHg)** | 70.9 ± 9.5 |  |
| **Q4**  **(+7 to +50 mmHg)** | 72.7 ± 10.1 |  | **Q4**  **(+14 to +36 mmHg)** | 69.0 ± 9.1 |  |

*Abbreviations: AIx, augmentation index; Aix@75, augmentation index adjusted for a standard heart rate of 75 bpm; c-f PWV, carotid femoral pulse wave velocity*; *DBP, diastolic blood pressure; HR, heart rate; Q, quartile; SBP; systolic blood pressure; SD, standard deviation*

ANOVA analysis showing the association between aortic stiffness (i.e., c-f PWV, AIx, and AIx@75) and central hemodynamics (central aortic blood pressure) stratified according to quartiles of orthostatic blood pressure reactions.

**Table S2. Comparison between individuals with and without manifest orthostatic hypotension, and the association with aortic stiffness and central hemodynamics.**

|  | **Fully adjusted model** | | |
| --- | --- | --- | --- |
|  | **OH** | **No OH** | **P-value** |
|  | **Mean ± SD** | **Mean ± SD** |  |
| **AORTIC STIFFNESS** | | | |
| **Direct (c-f PWV**, **m/s)** | 8.2 ± 1.6 | 7.5 ± 1.6 | 0.07 |
| **Indirect (Aix, mean%)** | 17.1 ± 14.8 | 8.4 ± 14.8 | 0.56 |
| **Indirect (Aix@75, mean%)** | 17.7 ± 15.0 | 8.4 ± 14.8 | 0.38 |
| **CENTRAL HEMODYNAMICS** | | | |
| **Central Aortic SBP (mmHg)** | 115.2 ± 17.7 | 103.3 ± 16.1 | 0.08 |
| **Central Aortic DBP (mmHg)** | 78.8 ± 10.5 | 72.5 ± 9.9 | 0.41 |

*Abbreviations: AIx, augmentation index; Aix@75, augmentation index adjusted for a standard heart rate of 75 bpm; BMI, body mass index; c-f PWV, carotid femoral pulse wave velocity*; *DBP, diastolic blood pressure; eGFR, estimated glomerular filtration rate; HR, heart rate; OH, orthostatic hypotension diagnosis; SBP; systolic blood pressure*

Linear regression analysis showing the association between aortic stiffness (c-f PWV, Aix, and Aix@75) and central hemodynamics (central aortic blood pressure) compared between individuals with and without manifest orthostatic hypotension.

Fully adjusted model adjusted for age, sex, BMI, eGFR, fasting glucose, current smoking antihypertensive medications, and supine SBP.

**Supplementary Figures.**

**Figure S1. Aortic stiffness and central hemodynamics measurements stratified according to quartiles of orthostatic *systolic* blood pressure reaction.**

Fig 1A. Augmentation index

Fig 1B. Augmentation index adjusted for heart rate

Fig 1C. Central aortic systolic blood pressure

Fig 1D. Central aortic diastolic blood pressure

**Figure S2. Aortic stiffness and central hemodynamics measurements stratified according to quartiles of orthostatic *diastolic* blood pressure reaction.**

Fig 2A. Augmentation index

Fig 2B. Augmentation index adjusted for heart rate

Fig 2C. Central aortic systolic blood pressure

Fig 2D. Central aortic diastolic blood pressure

**Figure S1. Aortic stiffness and central hemodynamics measurements stratified according to quartiles of orthostatic *systolic* blood pressure reaction.**


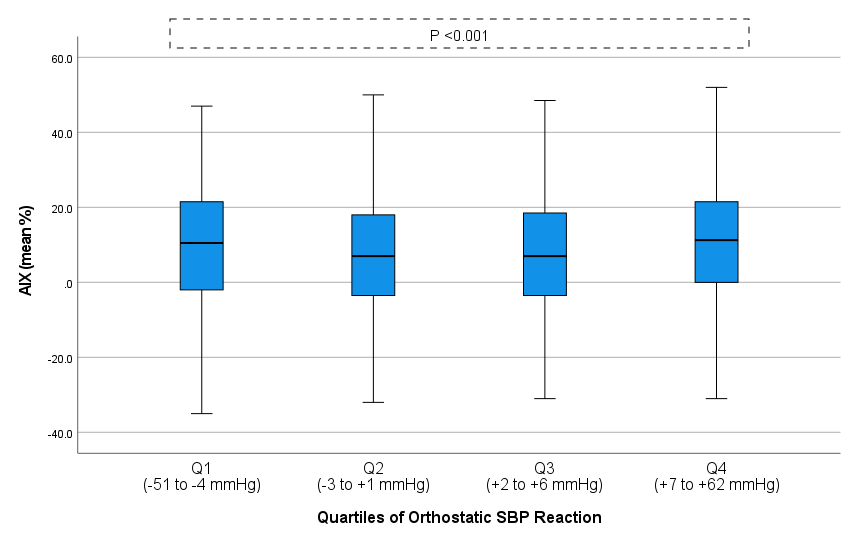


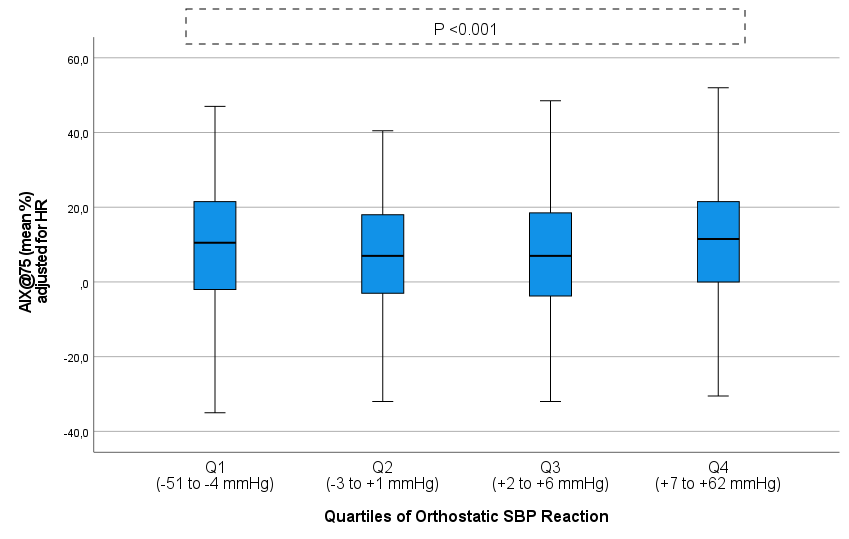


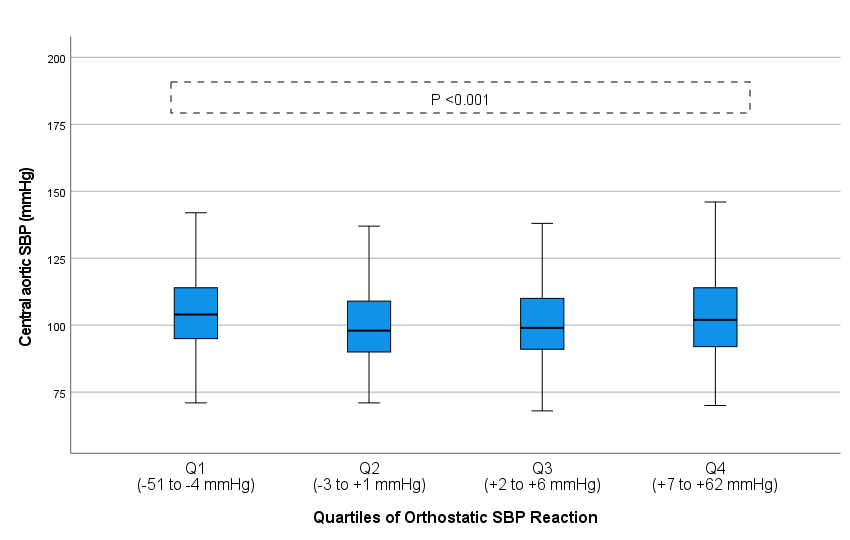


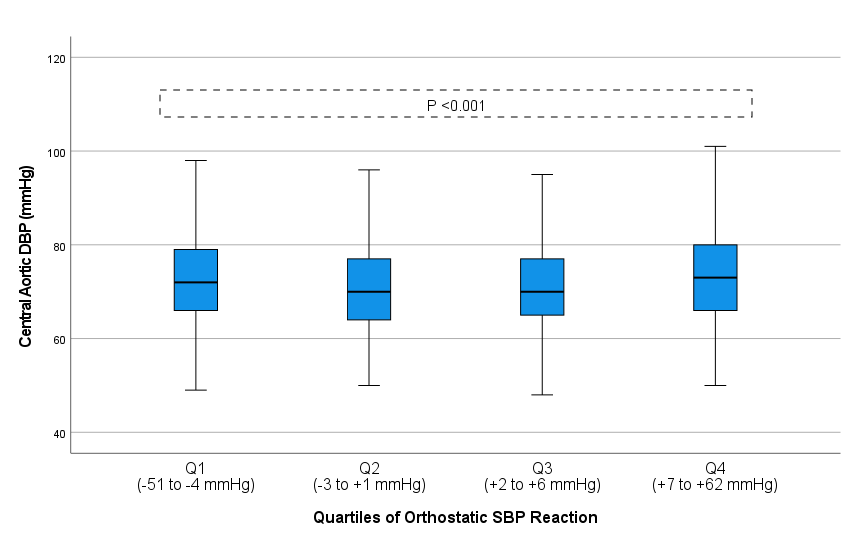


Boxplots illustrating aortic stiffness and central hemodynamics stratified according to quartiles of orthostatic systolic blood pressure reaction in the general population with reported ANOVA p-value. Fig 1A. Augmentation index, Fig 1B. Augmentation index adjusted for heart rate, Fig 1C. Central aortic systolic blood pressure, and Fig 1D. Central aortic diastolic blood pressure.

**Figure S2. Aortic stiffness and central hemodynamics measurements stratified according to quartiles of orthostatic *diastolic* blood pressure reaction.**


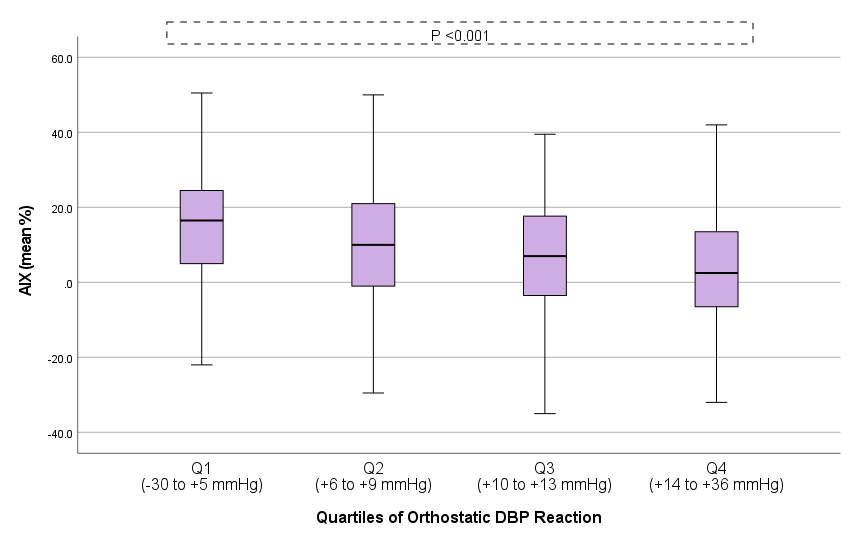


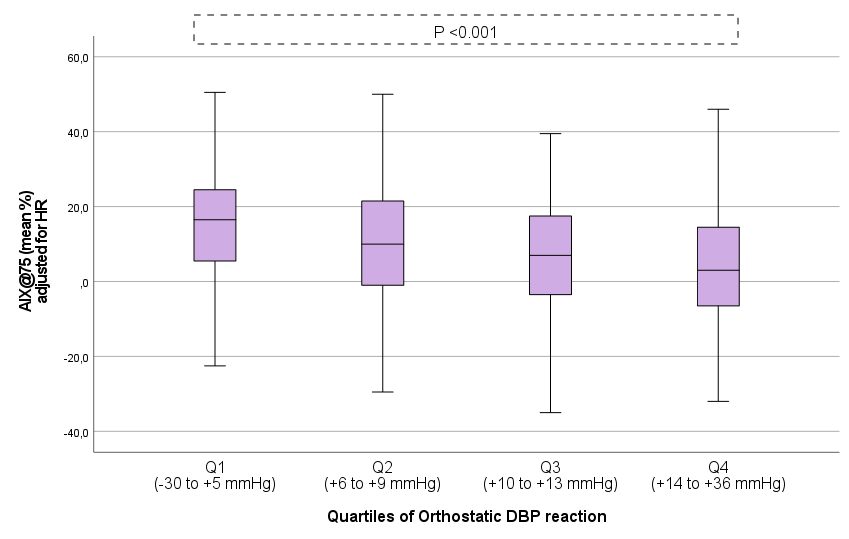


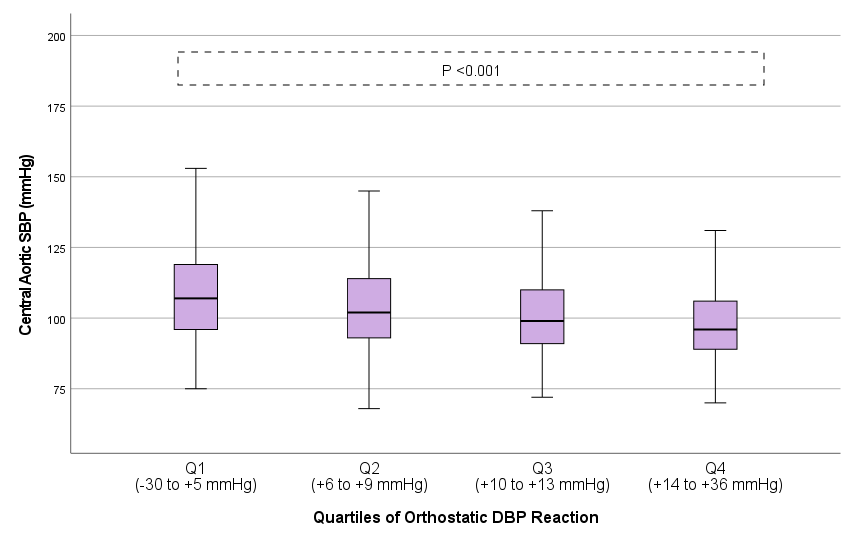


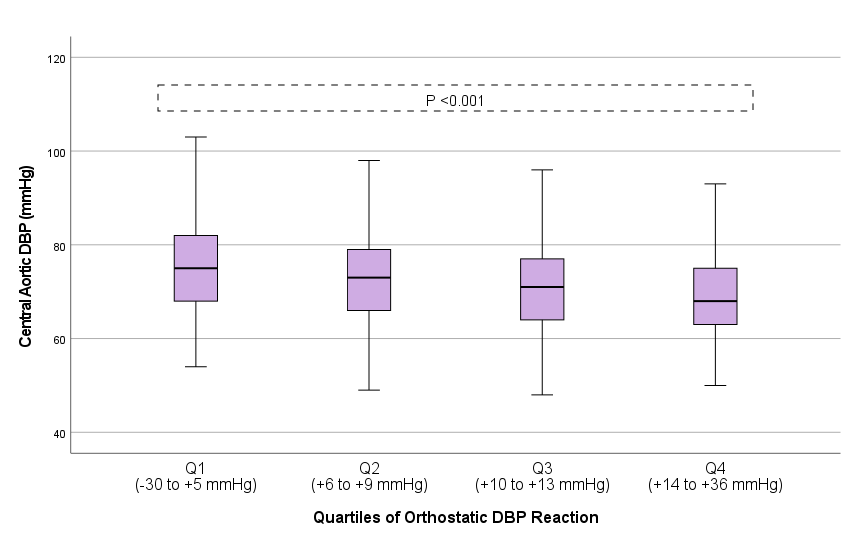


Boxplots illustrating aortic stiffness and central hemodynamics stratified according to quartiles of orthostatic diastolic blood pressure reaction in the general population with reported ANOVA p-value. Fig 2A. Augmentation index, Fig 2B. Augmentation index adjusted for heart rate, Fig 2C. Central aortic systolic blood pressure, and Fig 2D. Central aortic diastolic blood pressure.
